# Thermoregulated photothermal therapy for bone defects using graphene oxide hydrogel with mesoporous silica-derived phase-change nanocapsules

**DOI:** 10.1016/j.isci.2026.117650

**Published:** 2026-09-30

**Authors:** Qian Zhang, Shuang Zhao, Chenxi Shang, Yanan Jiang, Weiwei Zhang, Shuang Pan

**Affiliations:** 1 The First Affiliated Hospital of Harbin Medical University, School of Stomatology, Harbin Medical University, Harbin 150001, China; 2 State Key Laboratory of Frigid Zone Cardiovascular Diseases (SKLFZCD), Department of Pharmacology (State Key Laboratory-Province Key Laboratories of Biomedicine-Pharmaceutics of China), College of Pharmacy, Harbin Medical University, Harbin 150001, China; 3 Translational Medicine Research and Cooperation Center of Northern China, Heilongjiang Academy of Medical Sciences, Harbin, China

**Keywords:** photothermal therapy, thermal control, osteogenesis, hydrogel scaffold, encapsulation

## Abstract

The clinical translation of photothermal therapy for bone repair is hindered by the risk of thermal damage to surrounding healthy tissues due to uncontrolled high temperatures. We addressed this by integrating phase change materials (PCMs) into a photothermal platform, developing a hydrogel scaffold with temperature regulation. Polyethylene glycol (PEG) was encapsulated in mesoporous nanosilica (MSNs) to form nanocapsules, achieving 82.5% efficiency. This encapsulation prevents PEG leakage and enhances thermal regulation and bioactivity. Graphene oxide (GO) was integrated into an alginate-chitosan matrix, yielding a GO/chitosan scaffold containing PEG@MSNs nanocapsules. The scaffold exhibits a phase transition at 41.2°C and latent heat of 95.5 J/g. Characterization confirms its physicochemical properties, biocompatibility, and cell affinity meet bone repair requirements. *In vitro* mineralization assays, osteogenesis studies, and *in vivo* bone defect models demonstrated its efficacy in temperature-controlled photothermal bone regeneration. This integration of photothermal control and biological activity represents an advance in scaffold design.

## Highlights

• m-GO-PHSC enables precise temperature control at 41.2°C phase change for bone repair
• PEG@MSNs achieve 82.5% encapsulation and offer stable heat storage and cycling
• m-GO-PHSC demonstrates biocompatibility, controlled degradation, and systemic biosafety
• Bone defect models present 60.94% new bone formation to validate bone repair efficacy

## Graphical abstract

**Figure:**
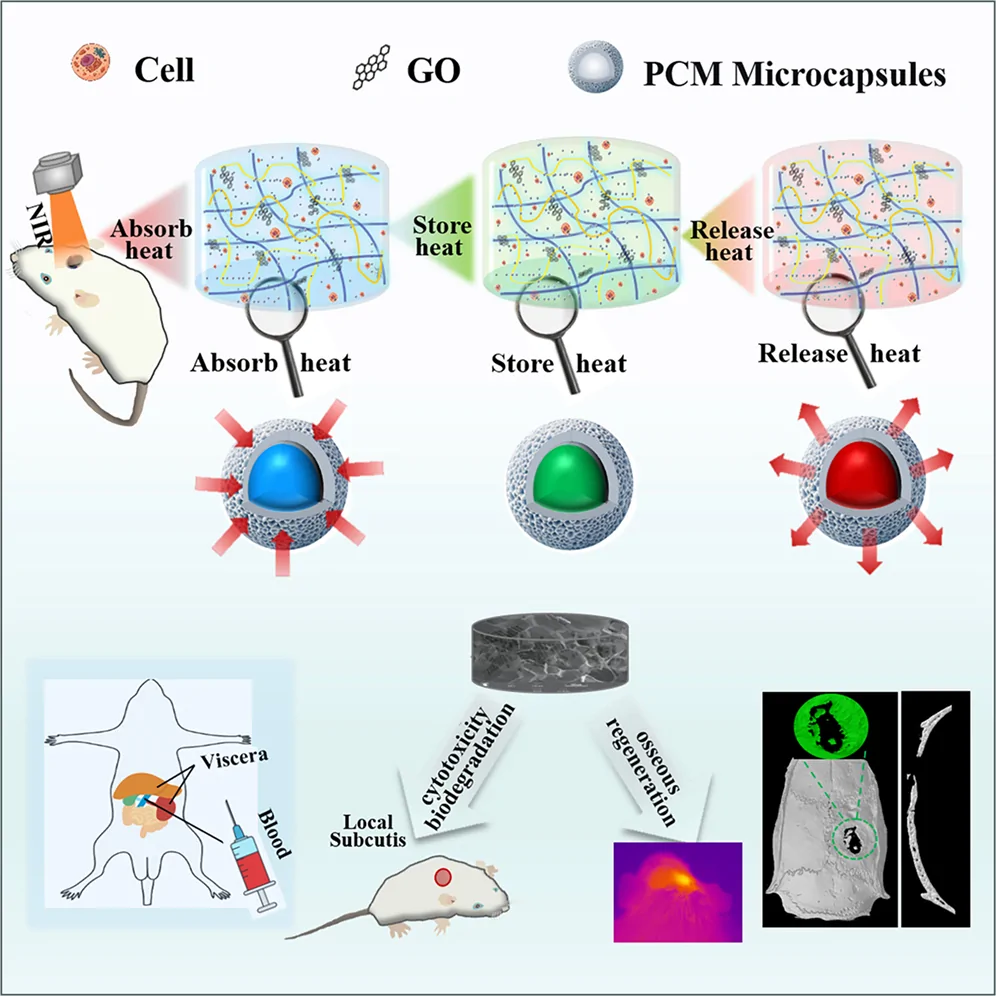

## Teaser

Biological sciences; Bioengineering; Tissue engineering; Biomaterials

## Introduction

Photothermal therapy (PTT) has emerged as a promising minimally invasive strategy that harnesses the light-to-heat conversion capabilities of photothermal agents—such as metallic nanostructures, small-molecule dyes, and carbon-based nanomaterials including graphene oxide (GO).^1^^,^^2^ GO is particularly attractive due to its exceptional photothermal conversion efficiency under 808 nm near-infrared (NIR) irradiation, a wavelength within the first biological transparency window that ensures deep tissue penetration with minimal absorption by hemoglobin and water, thereby enhancing biosafety.^3^^,^^4^ Its large specific surface area, abundant oxygen-containing functional groups, and excellent biocompatibility further make GO an ideal platform for constructing multifunctional therapeutic scaffolds.^5^^,^^6^

In bone tissue engineering, the incorporation of photothermal components into biomaterial scaffolds enables a synergistic treatment paradigm. Localized heat generated upon NIR irradiation can eradicate pathogenic bacteria while simultaneously stimulating osteogenic activity and promoting the secretion of bone-related factors, ultimately accelerating bone regeneration.^7^^,^^8^ Yanagi et al.^9^ provided compelling evidence that periodic photothermal stimulation delivered via carbon-nanotube-loaded hydrogels facilitated significant bone deposition in rat calvarial defects, underscoring the feasibility of leveraging photothermal cues to augment bone repair.

However, despite its potential, the clinical translation of PTT remains hindered by the inability to precisely regulate thermal dosage *in vivo*. Rapid heat accumulation during irradiation can exceed the therapeutic window (typically 40°C–43°C), causing protein denaturation, membrane disruption, and irreversible necrosis of surrounding healthy tissues. Conversely, abrupt thermal decay upon cessation limits sustained osteogenic signaling. The challenge is further compounded in bone tissue due to its heterogeneous thermal conductivity, low perfusion, and complex microarchitecture, which reduce heat dissipation and make conventional external feedback-based temperature control particularly unreliable. These factors highlight the urgent need for scaffolds capable of autonomous, real-time thermal self-regulation.

Phase-change materials (PCMs) can absorb or release large amounts of latent heat at nearly constant temperatures during phase transitions, providing an intrinsic mechanism for autonomous thermal regulation.^10^ Although originally developed for engineering heat-management systems, this unique thermal-buffering capability has led to their expanding use in biomedical applications such as controlled drug delivery, wearable thermotherapy, and implantable thermal management.^11^^,^^12^^,^^13^ A key advantage for PTT is that biocompatible PCMs, such as polyethylene glycol (PEG), offer tunable phase-transition temperatures.^14^ By matching the transition point to the therapeutic window of PTT, PCMs can stabilize the local tissue temperature and prevent overheating-induced cytotoxicity.^15^

Furthermore, recent advances in micro/nano-confinement and composite-structure design have effectively mitigated the challenges associated with solid-liquid transitions, thereby ensuring the structural integrity and long-term functionality of PCM-based biomedical scaffolds.^16^^,^^17^ Together, these properties, precise thermal buffering, biocompatibility, and structural stability, establish PCMs as a highly promising platform for achieving precise and safe temperature control during PTT.

To construct a composite hydrogel scaffold capable of integrating photothermal conversion with intrinsic thermal regulation, we employed a hydrated three-dimensional hydrogel network for its tissue-like mechanical properties, cytocompatibility, and capacity to host functional nanocomponents.^18^^,^^19^ Calcium-cross-linked sodium alginate (SA) was selected as the structural backbone, while chitosan (CS) was incorporated to provide osteogenic functions that are critical in bone defect environments often complicated by infection or chronic inflammation.^20^ GO acts as a photosensitizer and exhibits high biocompatibility. Its incorporation into the polymer matrix enhances the structural integrity of the composite scaffold and improves interfacial stability.^21^^,^^22^^,^^23^ PEG was chosen as the core thermoregulatory PCM. To prevent PEG leakage during its phase transition, we encapsulated PEG within mesoporous nanosilica (PEG@MSNs).^24^^,^^25^^,^^26^^,^^27^ These MSNs provide stable nanoscale confinement and can release bioactive silicon ions, which have been reported to promote osteogenesis.^28^^,^^29^

Here, we present a composite hydrogel scaffold termed m-GO-PHSC (chitosan‑modified graphene‑oxide‑incorporated hydrogel with mesoporous silica‑derived phase‑change nanocapsules [PHSC]) that integrates GO-mediated photothermal conversion with PEG@MSNs phase-change buffering within an SA/CS network. The MSN shell prevents PEG leakage while releasing osteogenic silicon ions, and the PEG core contributes to maintaining the local temperature within the 40°C–43°C therapeutic window under NIR irradiation. Comprehensive *in vitro* and *in vivo* evaluations (mineralization assays, gene expression, and rat calvarial defect models) demonstrate that this self-regulating scaffold achieves precise thermal dosage, avoids overheating-induced collateral damage, and markedly accelerates bone regeneration. This establishes a new paradigm of autonomous thermal homeostasis for PTT scaffolds. Figure 1 presents an overview of the conceptual design.

**Figure 1:**
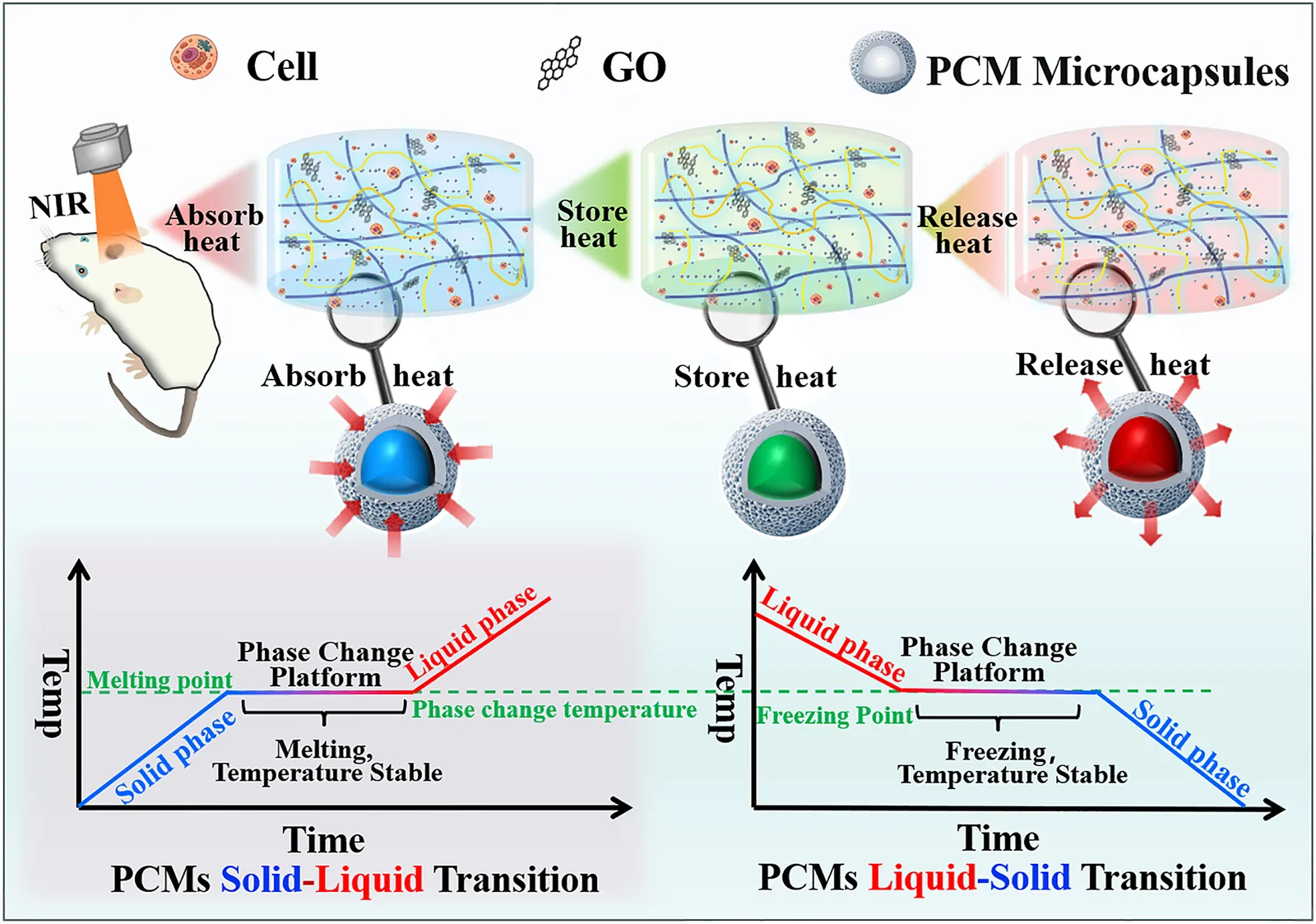
Schematic of the temperature control concept for phase change material

## Results

### Synthesis and thermal characterization of m-GO-PHSC

Figure 2 illustrates the schematic diagram of the preparation process for the m-GO-PHSC composite. Detailed synthesis procedures are provided in the STAR Methods section. To evaluate the form-stable encapsulation capability and thermal stability of PEG within MSNs, differential scanning calorimetry (DSC) was conducted for PEG@MSNs before and after thermal cycling, alongside the m-GO-PHSC hydrogel scaffold. Pure PEG1500 exhibited a phase transition temperature of 47.1°C and a latent heat of 166.3 J/g (Figure 3A). In contrast, PEG@MSNs showed a slightly lower phase transition temperature of 43.0°C and a latent heat of 137.2 J/g (Figure 3B), corresponding to an encapsulation efficiency of 82.5%. Thermal cycling tests further confirmed the stability of the form-stable PCM, with a phase transition temperature of 42.6°C and latent heat of 134.4 J/g after 100 cycles, indicating only a 2.04% decrease in latent heat (Figure 3C). Incorporation of PEG@MSNs into the hydrogel matrix yielded m-GO-PHSC, which exhibited a phase transition temperature of 41.2°C and a latent heat of 95.5 J/g (Figure 3D). The photothermal conversion performance, assessed via infrared thermal imaging under 1 W/cm^2^ NIR laser irradiation (Figure S1), revealed minimal heating for the GO-free *m*-PHSC (25.8°C–28.7°C in 2 min). In contrast, the GO-containing scaffolds m-GO-PHSC (PEG1500) and m-GO-PHSC (PEG6000) rapidly heated to 45.6°C and 48.1°C, respectively, under the same conditions (Figure 3E). Interestingly, a distinct temperature divergence occurred near the phase transition temperature of PEG1500-based m-GO-PHSC (41.2°C). Both scaffolds showed similar temperatures at 10 s (m-GO-PHSC (PEG1500): 32.1°C; m-GO-PHSC (PEG6000): 32.4°C). However, within 1 min, m-GO-PHSC (PEG1500) exhibited much more stable thermal behavior, maintaining 41.2°C, while the PEG6000-based scaffold rose to 44.9°C, resulting in a 3.7°C difference that persisted over this period. By 2 min, this temperature differential remained at 2.7°C (45.6°C vs. 48.1°C).

**Figure 2:**
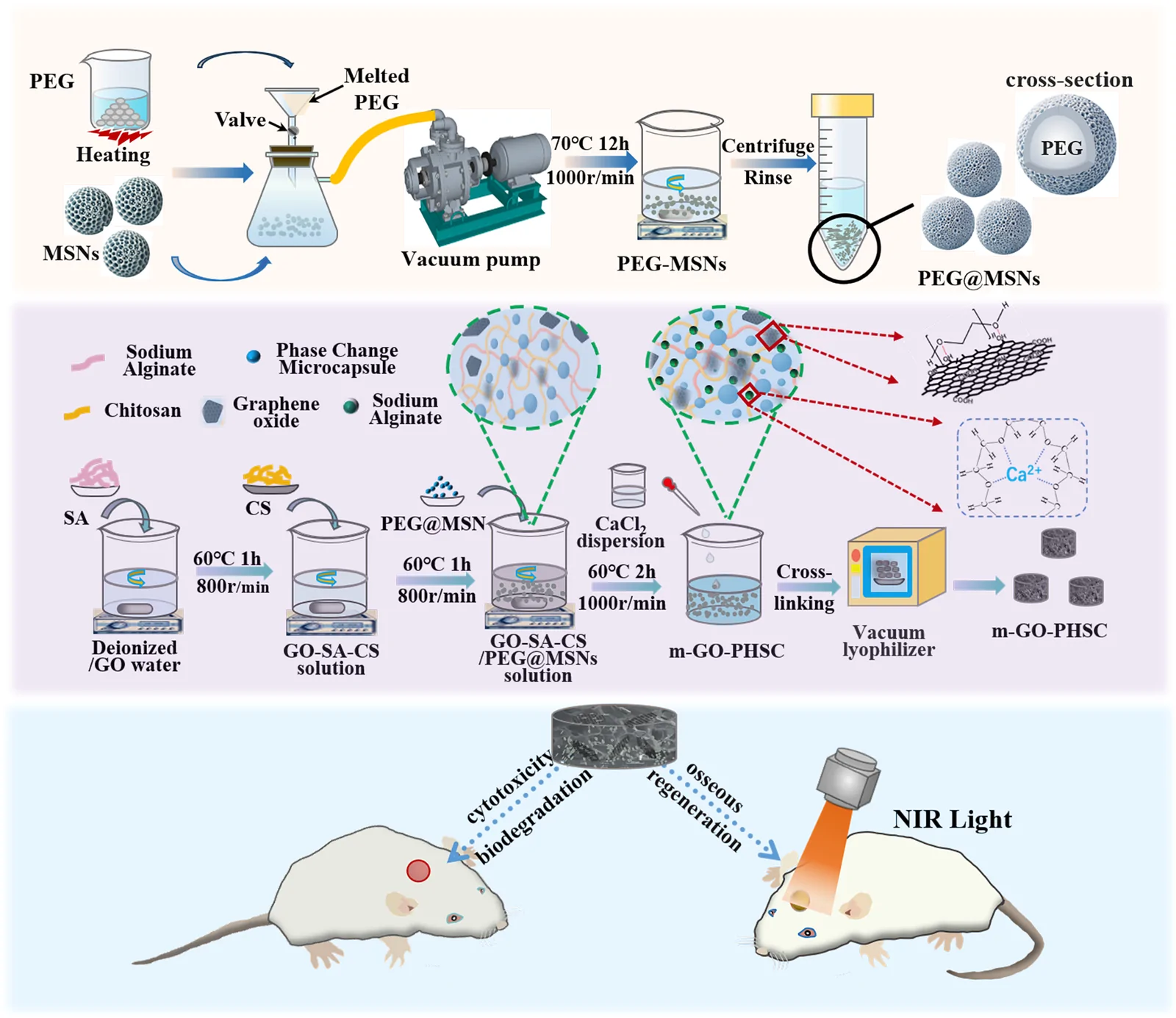
Schematic diagram of the preparation process of m-GO-PHSC, and its *in vivo* evaluation

**Figure 3:**
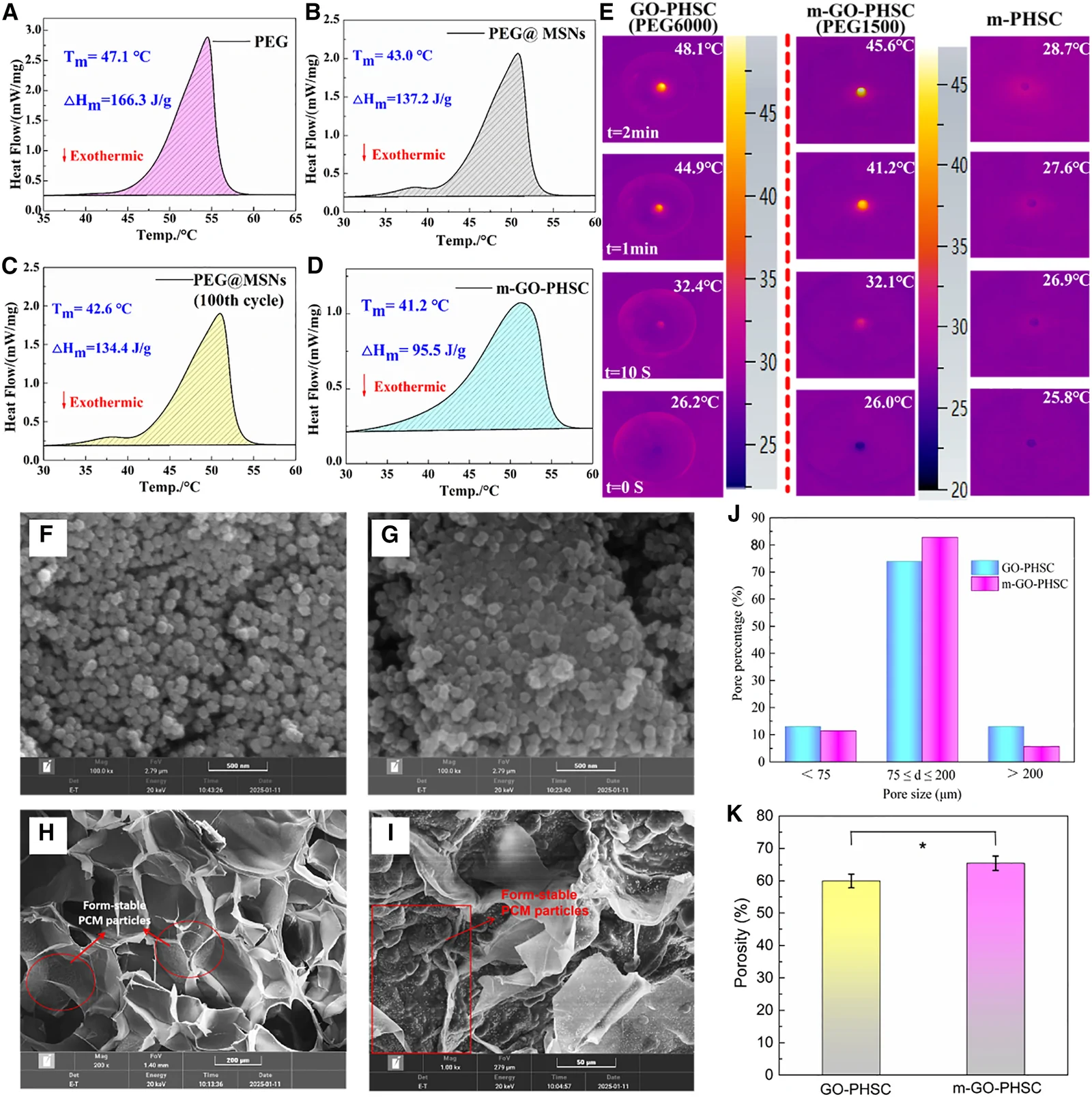
Thermal characterization of the composite phase change materials (A) DSC curve of pure PEG. (B) DSC curve of PEG@MSNs prior to thermal cycling. (C) DSC curve of PEG@MSNs subjected to 100 thermal cycles. (D) DSC curve of the m-GO-PHSC composite. (E) Infrared thermal images comparing the photothermal performance of m-GO-PHSC (PEG6000), m-GO-PHSC (PEG1500) and *m*-PHSC.SEM images and porosity analysis of PGE@MSNs and composite materials. (F and G) SEM image of the microstructure of MSNs and PGE@MSNs particles. (H and I) SEM images of the microstructure and internal pore structure of m-GO-PHSC, and (J) Pore volume distribution of GO-PHSC and m-GO-PHSC in the pore size range of 75–200 μm. (K) Porosity values of m-GO-PHSC measured by the ethanol displacement method. Statistical significance was determined by one-way ANOVA. *∗p <* 0.05,*∗∗p <* 0.01, *∗∗∗p <* 0.001. Scale bars: 500 μm in (F) and (G), 200 μm in (H), and 50 μm in (I). All experiments were independently repeated three times. DSC measurements were performed under N_2_ at 5°C/min from −10°C to 60°C; SEM specimens were sputter-coated with gold.

### Chemical composition, microstructure, and degradation behavior

Attenuated total reflectance (ATR) spectroscopy was employed to analyze the molecular vibrations and interactions within PEG@MSNs and m-GO-PHSC hydrogel scaffold (Figure S2). The spectrum of PEG@MSNs exhibited characteristic absorption peaks of both PEG (such as the CH_2_ stretching vibration at 2,880 cm^−1^) and MSNs (such as the Si-*O*-Si asymmetric stretching vibration near 842 cm^−1^). A distinct baseline elevation observed at the latter position indicates the successful encapsulation of PEG within the mesoporous silica network. For the CS-based composite PCMs (CS-PHSC, CS-GO-PHSC, and m-GO-PHSC), the characteristic absorption peaks of CS (around 1,600 and 1,425 cm^−1^) consistently appeared in their spectra, confirming the successful incorporation of CS. Notably, upon the introduction of GO, the relative intensities of the characteristic peaks near 1,600, 1,425, and 1,070 cm^−1^ in the CS-GO-PHSC spectrum were significantly enhanced. In the ATR spectrum of GO, the peak at 1,032 cm^−1^ represents C-O stretching vibration, the peak at 1,406 cm^−1^ corresponds to C-OH bending vibration, and the peak at 1,617 cm^−1^ represents C=C skeletal vibration. For SA, the peaks at 1,600 and 1,404 cm^−1^ represent asymmetric stretching of COO^−^, indicating the presence of carboxylate groups. For PEG, the 2,880 cm^−1^ peak corresponds to symmetric -CH_2_- stretching, while peaks at 1,465 and 1,340 cm^−1^ represent -CH_2_- bending and -CH_3_- symmetric deformation vibrations, respectively. C-O stretching is observed at 1,102 cm^−1^, with in-plane C-O and C-*O*-H deformations at 945 cm^−1^.^30^ For MSNs, a characteristic absorption appears at 2,926 cm^−1^, with Si-*O*-Si asymmetric and symmetric stretching vibrations at 1,057 and 800 cm^−1^,^31^ respectively. The Fourier transform infrared (FTIR) spectrum of PEG@MSNs displayed characteristic PEG absorption bands at 2,880 (CH_2_ symmetric stretching), 1,465 (CH_2_ bending), 1,340 (CH_3_ deformation), 950 (C-O stretching), and 842 cm^−1^ (C-*O*-C vibration). The peak observed at 2,939 cm^−1^ corresponds to the CH_2_ stretching vibration of nano-MSNs (2,926 cm^−1^). Notably, the absorption feature at 842 cm^−1^ exhibited a distinct baseline elevation, which can be attributed to the overlapping Si-*O*-Si asymmetric stretching vibrations from the nano-MSNs. These spectroscopic results confirm the successful shape stabilization of PEG via nano-MSNs encapsulation, providing clear evidence of physical interactions between the components. In the CS spectrum, the peak at 3,049 cm^−1^ corresponds to the stretching vibration of -NH_2_, while the absorption peak at 1,585 cm^−1^ indicates the presence of an amide group, attributed to the combined effects of N-H bending and C-N stretching vibrations. Additionally, the absorption peak at 1,138 cm^−1^ is associated with the pyran ring stretching vibrations in CS and the C-NH_2_ (amino) group. In the ATR spectra of CS-PHSC, CS-GO-PHSC, and m-GO-PHSC, characteristic absorption peaks at 1,600 and 1,425 cm^−1^ consistently appeared, confirming the successful incorporation of CS. Upon the introduction of GO, the relative intensities of the absorption peaks at 1,600, 1,425, and 1,070 cm^−1^ in the CS-GO-PHSC spectrum significantly increased compared to those in GO-PHSC, suggesting the formation of hydrogen bonding interactions between GO and PHSC.^32^^,^^33^ In conclusion, the results confirm that the integration of GO and CS-PHSC was primarily driven by physical interactions rather than chemical reactions. Compared to the 825 cm^−1^ absorption peak in CS-GO-PHSC, the corresponding peak in m-GO-PHSC is broader and more intense. This broadening is attributed to the Si-*O*-Si stretching vibration of nano-MSNs at 800 cm^−1^, which also exhibits a leftward shift in the spectrum, indicating interactions within the composite material. An ideal scaffold material should mimic the morphology and structure of natural bone to facilitate effective bone regeneration.^34^ The presence of pores within the scaffold is critical for successful bone formation, as they promote cell infiltration, new tissue growth, nutrient diffusion, vascularization, and metabolic waste removal.^35^ To analyze the microstructure and internal porosity of m-GO-PHSC hydrogel scaffold, SEM was employed.

The microscopic morphology of MSNs and PEG@MSNs is shown in Figures 3F and 3G. Pristine MSNs displayed a uniform spherical morphology with an average particle diameter of approximately 100 nm and minimal aggregation. PEG@MSNs maintained the nanoscale features of the original MSNs; however, slight aggregation was observed. The microstructure of the m-GO-PHSC hydrogel scaffold is illustrated in Figures 3H and 3I. The scaffold exhibited a highly porous, interconnected three-dimensional architecture with uniform pore distribution, forming a structurally stable framework. The pore walls displayed well-defined structural features. Moreover, form-stable PCM particles (PEG@MSNs) were homogeneously dispersed along the pore walls and matrix surface, confirming their effective integration into the scaffold. Image-based pore size quantification (Figure 3J) revealed that 82.9% of scaffold pores fell within the ideal 75–200 μm range, with only 11.4% below 75 μm and 5.7% above 200 μm. The porosity of m-GO-PHSC, determined by ethanol displacement (Figure 3K), was 65.4% ± 2.2%. In addition to structural considerations, the m-GO-PHSC scaffold exhibited a controlled, biphasic degradation profile over 42 days in phosphate-buffered saline (PBS) (Figure S3), with an initial rapid phase followed by stabilization. Within the first 7 days, mass loss reached 23.3% ± 1.21%, with 10.67% ± 0.78% occurring within the first 24 h. Thereafter, degradation slowed, with only 9.33% additional loss over the next five weeks (7–42 days), reaching 32.63% ± 1.91% at six weeks.

### Bioactivity evaluation

The cytocompatibility of m-GO-PHSC was assessed using mouse bone marrow mesenchymal stem cells (mBMSCs) cultured with hydrogel extracts via CCK-8 analysis and live/dead staining (Figure 4A). m-GO-PHSC supported markedly enhanced cell proliferation, exhibiting viabilities of 111.2% ± 8.1% (day 1) and 114.0% ± 7.9% (day 2), significantly exceeding the ∼80% observed for GO-PHSC (Figure 4C). Live/dead staining (Figure 4B) corroborated these findings, revealing substantially greater viability in the m-GO-PHSC group than in GO-PHSC and the normal culture (NC) control after 2 days. Transwell assays demonstrated a pronounced chemotactic effect of scaffold extracts on mBMSCs (Figures 4D–4F). m-GO-PHSC elicited the strongest pro-migratory response, with migration rates of 846.7 ± 15.2 cells at 24 h and 1,313.3 ± 40.4 cells at 48 h, significantly exceeding both GO-PHSC (433.3 ± 15.3 cells and 620 ± 10 cells) and NC (556.7 ± 20.8 cells and 870 ± 20 cells) (*p* < 0.001).

**Figure 4:**
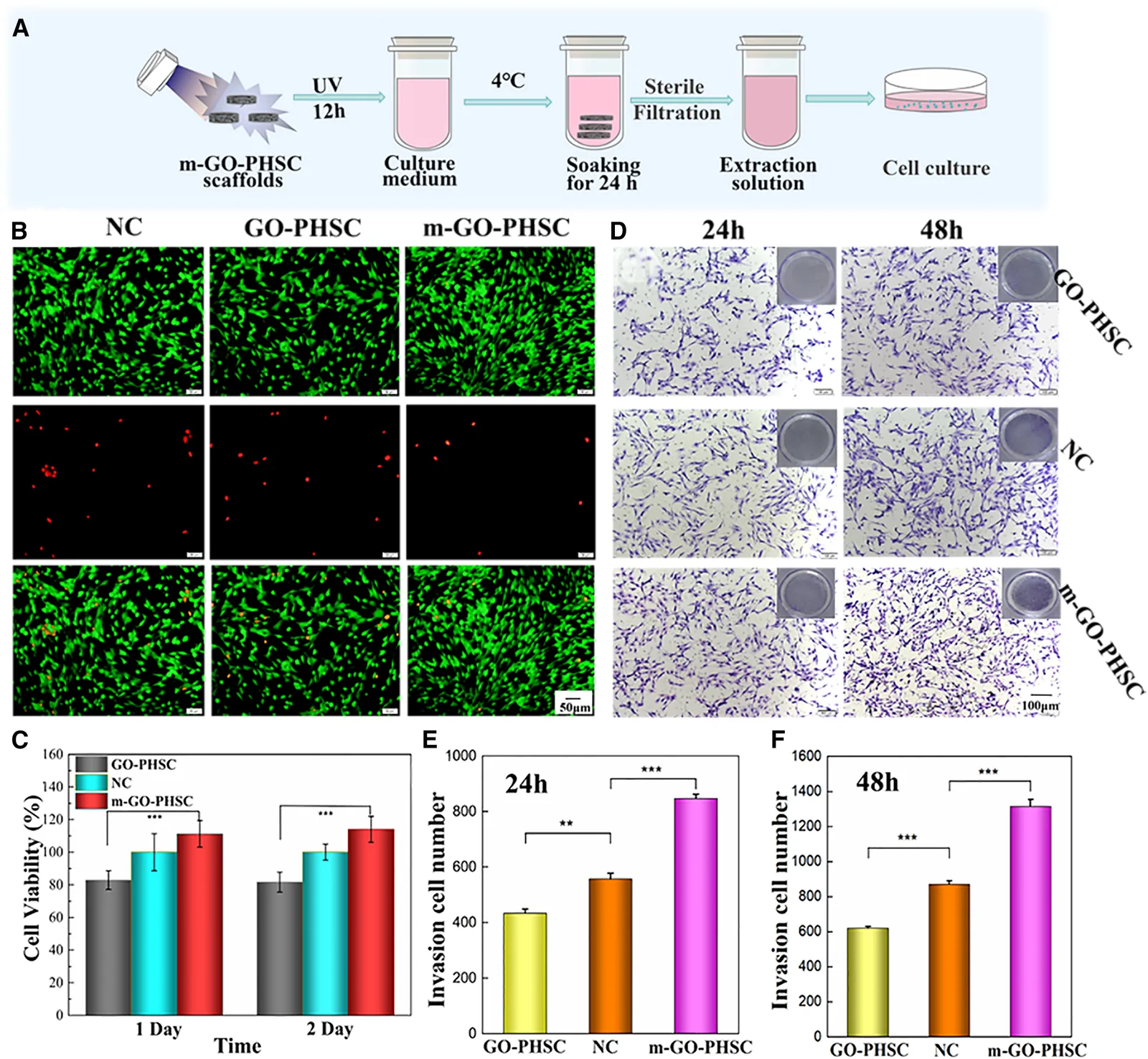
*In vitro* evaluation of the biological effects of composite extracts on mBMSCs (A) Extract preparation: schematic of the m-GO-PHSC extract preparation. (B and C) Cell viability. (B) Live/dead staining images of mBMSCs after 2-day culture (green: live cells; red: dead cells). (C) Quantitative cell viability measured by CCK-8 assay over 2 days. (D–F) Cell migration, (D) Representative images from the Transwell migration assay at 24 and 48 h. (E and F) Quantitative analysis of migrated cells at (E) 24 h and (F) 48 h. Statistical significance was determined by one-way ANOVA. *∗p <* 0.05,*∗∗p <* 0.01, *∗∗∗p <* 0.001. Scale bars: 50 μm in (B) and 100 μm in (D). All experiments were independently repeated three times. Quantitative data (CCK-8 and Transwell migration assays) were obtained from *n* = 3 technical replicates, each with three parallel wells, and are presented as mean ± SD. Representative images from one experiment are shown for live/dead staining and Transwell assays.

### *In vitro* mineralization and osteogenic induction

The osteogenic capacity of the CS-modified m-GO-PHSC hydrogel scaffold was systematically evaluated through mineralization and gene expression analyses (Table 1). After 14 days of culture, Alizarin Red S (ARS) staining demonstrated a clear hierarchical enhancement in matrix mineral deposition across the four groups (Figures 5A–5E). Quantitative analysis revealed mineralization rates of 1.0% ± 0.03% (control), 20.7% ± 0.31% (osteogenic medium, OCM), 29.95% ± 1.60% (GO-PHSC), and 43.83% ± 3.69% (m-GO-PHSC), with both scaffold groups significantly outperforming the standard OCM (*p* < 0.01). To further substantiate the osteoinductive capability of m-GO-PHSC and evaluate potential photothermal enhancement, quantitative PCR was performed to assess the expression of osteogenesis-related genes (HSP70, ALP, bone morphogenetic protein‑2 (BMP‑2), and RUNX-2) after 7 and 14 days of culture (Figures 5F–5I). On day 7, the m-GO-PHSC + light group showed numerically higher expression of HSP70 (3.72% ± 1.11%) and ALP (4.39% ± 0.50%) compared with the m-GO-PHSC without light group (2.56% ± 0.41%, 3.57% ± 0.33%) and the OCM group (1.59% ± 0.42%, 2.71% ± 0.29%). However, no statistically significant difference in HSP70 expression was observed between the +light group and the group without light (*p* > 0.05). By day 14, the differences became more pronounced. The m-GO-PHSC+ light group showed the highest transcriptional levels of three genes (HSP70: 9.93% ± 0.47%; ALP: 15.16% ± 2.11%; BMP-2: 13.59% ± 2.05%), which were significantly higher than those in both the non-irradiated m-GO-PHSC group and the OCM group (*p* < 0.05). Meanwhile, m-GO-PHSC without light remained significantly stronger than OCM across all markers (*p* < 0.05).

**Table 1:** Specific sequences

Gene	Primer sequence (5′ to 3')
Runx2-F	TGGCCGGGAATGATGAGAAC
Runx2-R BMP2-F BMP2-R ALP-F ALP-R	CTCCGGCCCACAAATCTCAG CAGCCCGATCACCTCTCTTC GCTTTTCTTCGCCTCCTCCT GGA CAG GAC ACA CAC ACA CA CAA ACA GGA GAG CCA CTT CA
HSP70-F HSP70-R GAPDH-F GAPDH-R	ATCAAGCGCAACTCCACCAT CCTCGTACACCTGGATCAGC GGAGCGAGATCCCTCCAAAAT GGCTGTTGTCATACTTCTCATGG

**Figure 5:**
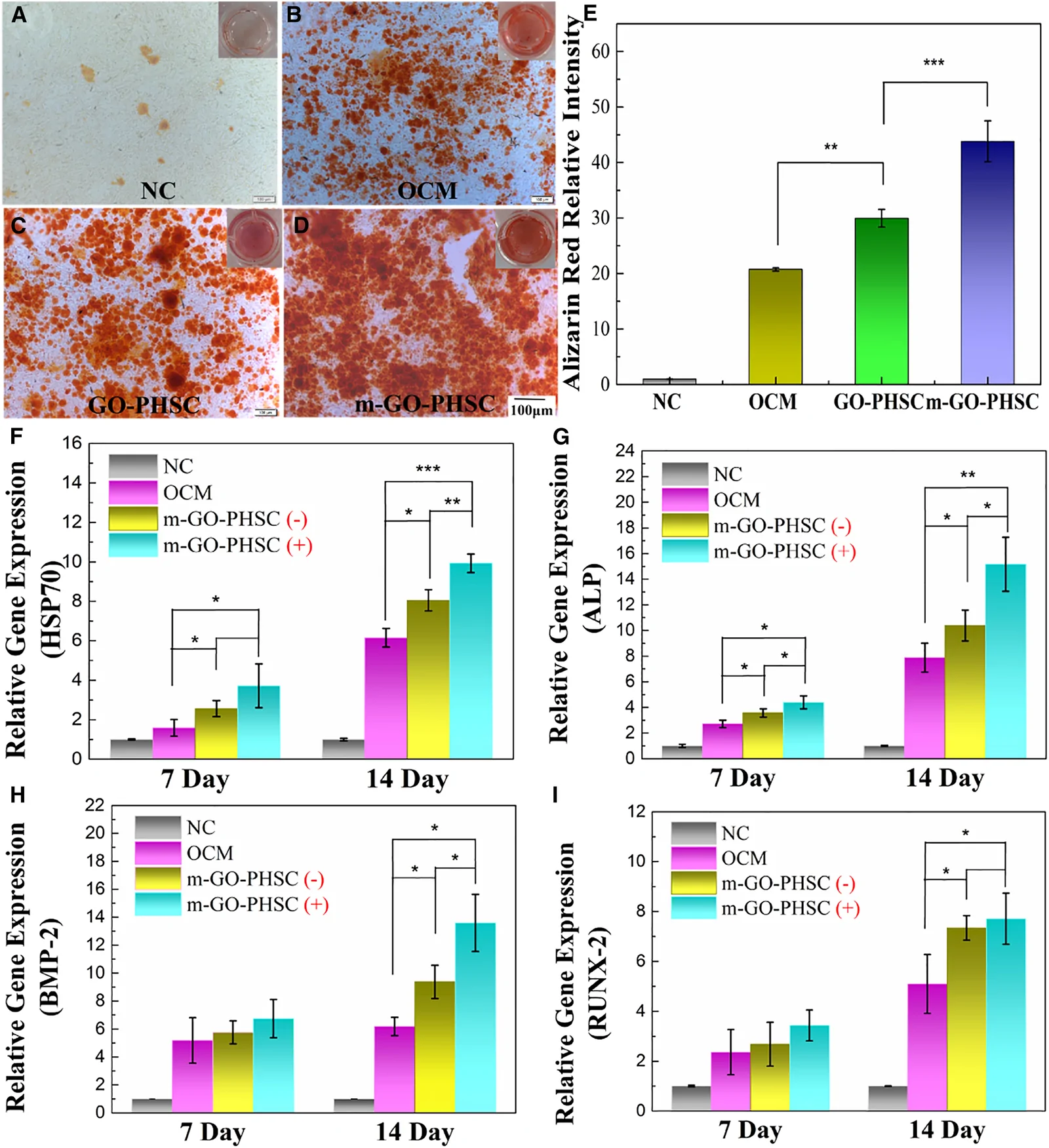
Osteogenic differentiation of mBMSCs induced by GO-PHSC and m-GO-PHSC extracts (A–D) ARS staining of cells cultured in NC (complete medium), OCM (osteogenic medium), GO-PHSC, and m-GO-PHSC extracts, respectively. (E) Semi-quantitative analysis of ARS staining. (F–I) Relative mRNA expression levels of osteogenic marker genes (HSP70, ALP, BMP-2, and RUNX-2) in OCM, m-GO-PHSC (without NIR), and m-GO-PHSC + NIR (808 nm, 1 W/cm^2^, 2 min per session, every 72 h) groups at day 7 and 14, normalized to GAPDH. Statistical significance was determined by one-way ANOVA. ∗*p* < 0.05, ∗∗*p* < 0.01, ∗∗∗*p* < 0.001. Scale bars, 100 μm in (A–D). All experiments were independently repeated three times. Quantitative data are from *n* = 3 technical replicates and presented as mean ± SD. Representative images from one experiment are shown for ARS staining.

### *In vivo* thermoregulation

To evaluate the *in vivo* thermoregulation capability of m-GO-PHSC, scaffolds were implanted into bone defect sites, and photothermal conversion under NIR irradiation was monitored. Three groups were assessed: blank (no scaffold), m-GO-PHSC (PEG6000) as control group, and m-GO-PHSC group (Figure 6). The scaffold-implanted groups exhibited significant temperature increases compared to the blank group. The *in vivo* photothermal profiles revealed that after 1 min of irradiation, the Blank, Control, and m-GO-PHSC groups reached temperatures of 35.9°C, 40.2°C, and 39.1°C, respectively. After 2 min, the temperatures rose to 36.3°C, 46.3°C, and 42.8°C, respectively. Following a 3-min recovery period without irradiation, the temperatures decreased to 35.6°C, 37.9°C, and 39.7°C, respectively. Notably, the m-GO-PHSC group exhibited slower heating and cooling rates, maintaining temperatures within the optimal osteogenic window throughout irradiation. In contrast, the control group exceeded this range.

**Figure 6:**
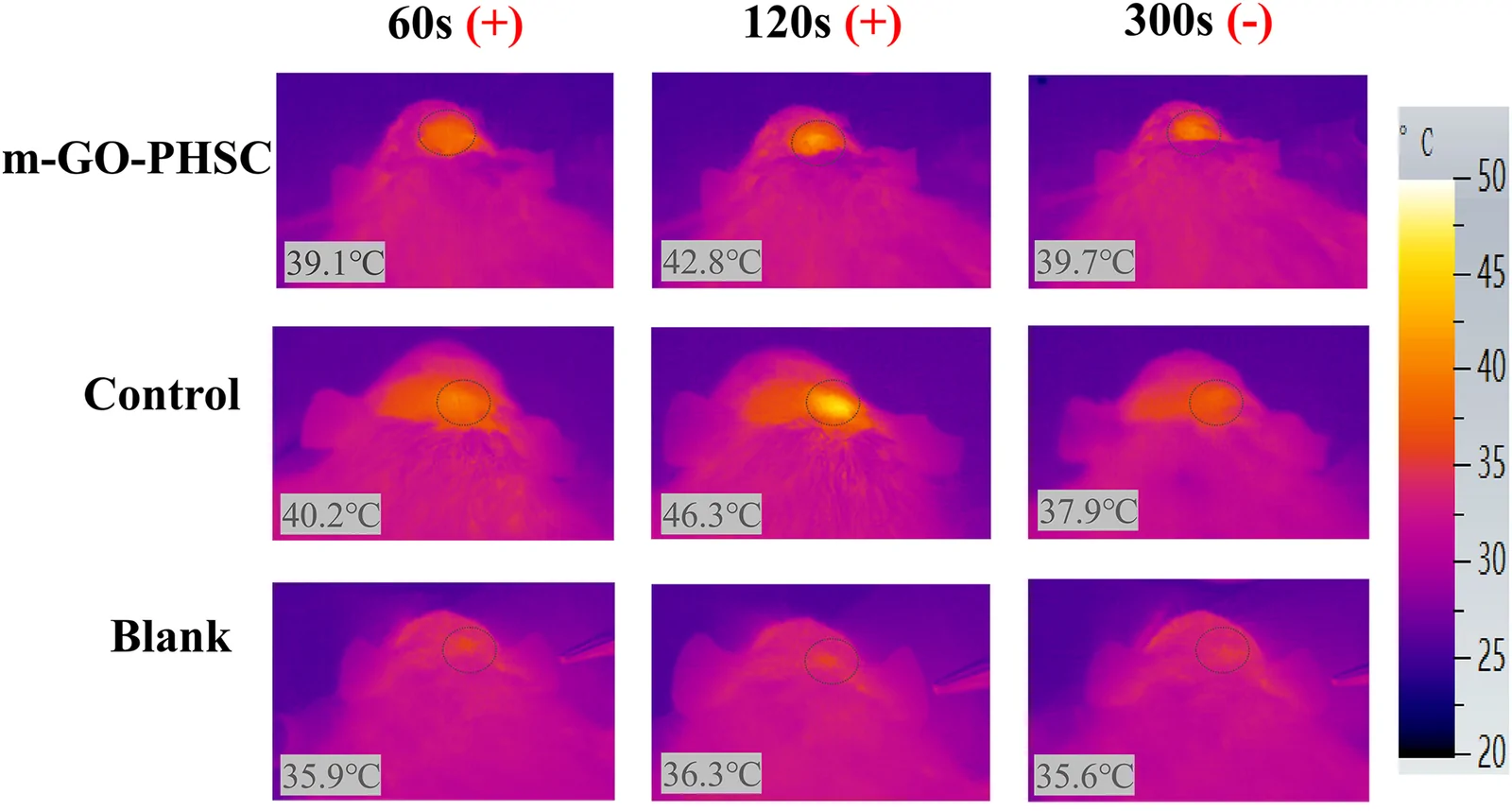
*In vivo* temperature control effect evaluation of hydrogel scaffolds under NIR irradiation (808 nm, 1.0 W/cm^2^, 2 min; *n* = 3 rats/group) Temperature was monitored by infrared thermal imaging. Blank group: no scaffold material implanted; control group: m-GO-PHSC(PEG6000) photothermal hydrogel scaffold implanted; m-GO-PHSC group: m-GO-PHSC hydrogel scaffold implanted. Temperatures were recorded at 60 s, 120 s, and after 3 min recovery.

### *In vivo* degradation performance and biocompatibility evaluation

To investigate *in vivo* behavior, the hydrogel was subcutaneously implanted in Sprague-Dawley (SD) rats (Figures 7A and 7B). Four weeks post-implantation, the hydrogel exhibited gradual volume reduction while maintaining its overall ellipsoidal shape and structural integrity, with no noticeable fragmentation. Local tissue responses were evaluated via histological analysis of the implantation sites (Figure 7C). The negative control (NC) group, which underwent the surgical procedure without hydrogel implantation, established a baseline for normal tissue morphology and inflammatory status. H&E staining of the NC group confirmed intact muscle architecture with nearly no inflammatory infiltration. In comparison, the GO-PHSC hydrogel induced mild chronic inflammation, evidenced by moderate inflammatory cell infiltration. Remarkably, the m-GO-PHSC hydrogel exhibited minimal inflammatory infiltration, comparable to the NC group, with no statistically significant difference. Comprehensive organ dissection and histological examination were conducted to evaluate systemic biosafety (Figure 7D). Major organs—including heart, liver, spleen, lungs, and kidneys—showed no structural abnormalities, inflammation, necrosis, or fibrosis in either hydrogel-implanted group compared with controls. Consistently, serum biochemical analysis revealed that all parameters, including blood urea nitrogen (BUN), alanine aminotransferase (ALT), creatinine (CREA) and albumin (ALB), remained within normal ranges (Figure 7E).

**Figure 7:**
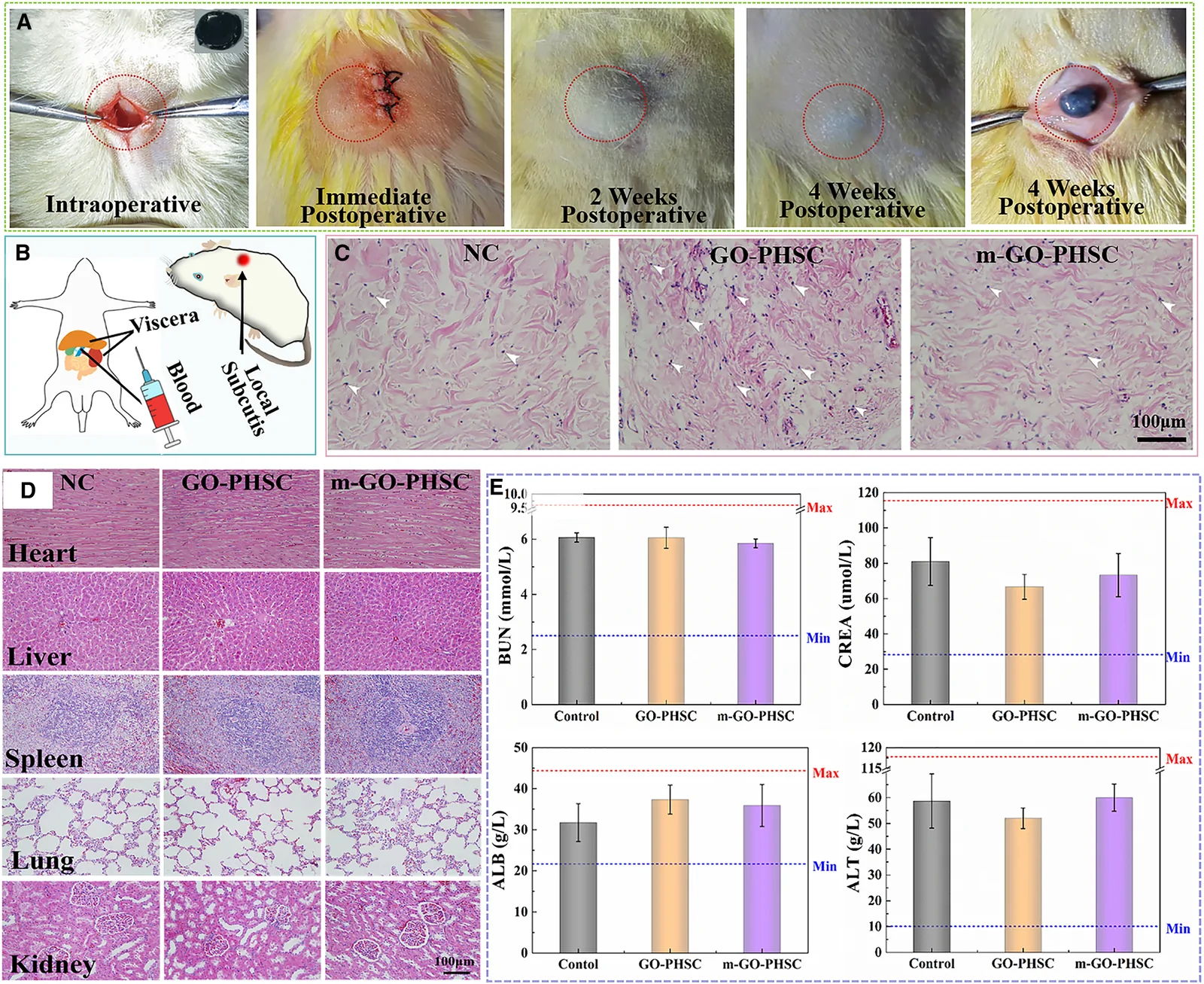
*In vivo* degradation and biocompatibility assessment of m-GO-PHSC hydrogel (A) Schematic of the subcutaneous implantation model in rats. (B) Local tissue response and systemic biocompatibility evaluation at predetermined time points. (C) Histological analysis (H&E staining) of the subcutaneous tissue surrounding the implant after 1 month (white arrows: inflammatory cells). (D) H&E staining of major visceral organs (heart, liver, spleen, lungs, and kidneys) at 2 months post-implantation. (E) Serum biochemistry at 2 months: BUN, CREA, ALT, ALB. Statistical significance was determined by one-way ANOVA. ∗*p* < 0.05, ∗∗*p* < 0.01, ∗∗∗*p* < 0.001. Scale bars, 100 μm in (C and D). All data are from an exploratory cohort of *n* = 3 rats per group and are presented as mean ± SD.

### *In vivo* osteogenic evaluation

The osteogenic efficacy of the m-GO-PHSC hydrogel scaffold was assessed using a 5 mm rat calvarial defect model (Figure 8A). Micro-computed tomography (CT) scans were performed at 1 and 2 months post-implantation (Figures 8B–8G) to quantify new bone formation. Analysis of the bone volume/total volume (BV/TV) ratio (Figures 8H and 8I) revealed significantly enhanced bone regeneration in the m-GO-PHSC + photothermal irradiation group compared with both the m-GO-PHSC without irradiation and the no implantation (NC) groups at both time points. Consistently, histomorphometric evaluation of new bone area (Figures 8J and 8K) showed that the m-GO-PHSC + irradiation group achieved 45.96% ± 2.55% at 1 month and 60.94% ± 3.29% at 2 months, markedly higher than the m-GO-PHSC without irradiation (15.85% ± 0.93% and 37.25% ± 2.94%, respectively, *p* < 0.001) and NC groups (11.43% ± 0.87% and 20.22% ± 1.46%, respectively; *p* < 0.001). Histological analyses at 8 weeks using H&E and Masson’s trichrome staining (Figures 8L–8N) revealed clear differences in bone regeneration among groups. The NC group exhibited only a thin marginal bony bridge, with the defect center predominantly filled with fibrous tissue, indicating minimal intrinsic repair. In contrast, both m-GO-PHSC scaffold groups showed substantial new bone formation surrounding the defect, with the m-GO-PHSC + irradiation group presenting the most extensive regeneration. Newly formed bone displayed tight integration with adjacent connective tissue, abundant osteoid matrix, and visible scaffold remnants.

**Figure 8:**
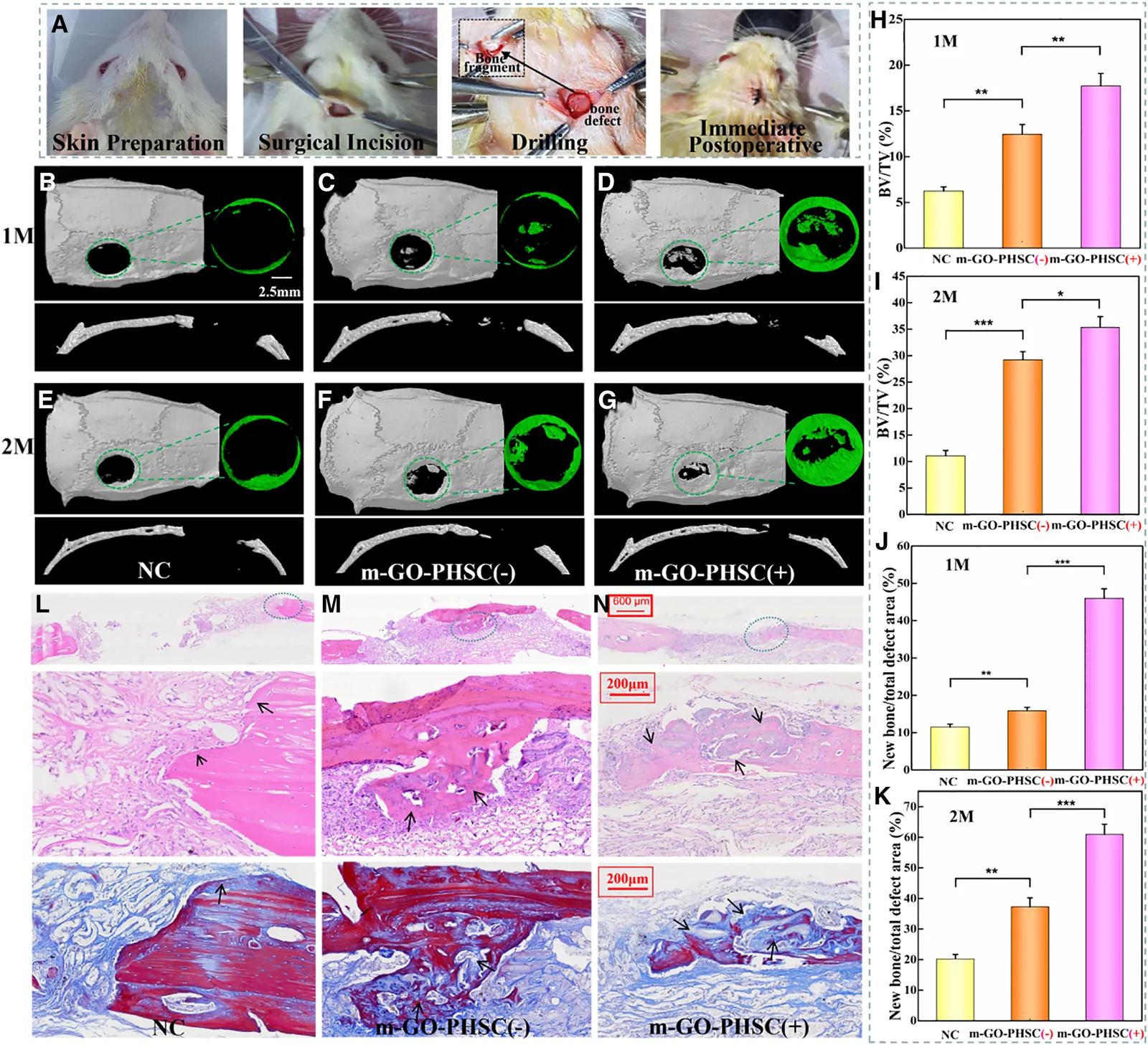
Bone regeneration in rat calvarial defects implanted with m-GO-PHSC hydrogel (A) Schematic of critical-sized calvarial defect creation and scaffold implantation. (B–G) Representative 3D reconstructions (top images) and coronal micro-CT sections (bottom images) at 1 month (B–D) and 2 months (E–G) post-operation. (B and E) Empty defect (NC), (C and F) m-GO-PHSC without NIR irradiation, and (D and G) m-GO-PHSC with NIR irradiation (808 nm, 1.0 W/cm^2^, 2 min per session, every 72 h). (H–K) Quantitative histomorphometry of bone volume fraction (BV/TV, %) and new bone area (%) at 1 and 2 months. (L–N) Histological sections (H&E and Masson’s trichrome) at 8 weeks, (L) NC, (M) m-GO-PHSC without NIR, and (N) m-GO-PHSC with NIR. Statistical significance was determined by one-way ANOVA. ∗*p* < 0.05, ∗∗*p* < 0.01, ∗∗∗*p* < 0.001. Scale bars: 2.5 mm in (B–G), 600 μm and 200 μm in (L–N). Data are from *n* = 3 rats per group, presented as mean ± SD. All animal experiments were performed in a single cohort, individual data points represent each animal.

## Discussion

This study aimed to develop a photothermal and thermoregulatory composite hydrogel scaffold (m-GO-PHSC) for bone regeneration and to evaluate its multifaceted performance, from material properties to *in vivo* efficacy. The comprehensive results presented earlier underscore the successful fabrication of a scaffold with a combination of favorable thermal regulation, biocompatibility, and osteoinductive potential. The discussion that follows interprets these key findings, situates them within the context of prior work, and explores their significance for the field of bone tissue engineering.

### Material design and thermal regulation mechanism

The synthesis and characterization data confirm the successful construction of the m-GO-PHSC scaffold with its designed multifunctionality. The high specific surface area and abundant internal porous channels of MSNs were effectively utilized to microencapsulate PEG, enhancing its thermal performance and stability.^36^ DSC results demonstrated that PEG@MSNs possessed excellent form-stable encapsulation and thermal reliability, with minimal latent heat loss (2.04%) after 100 thermal cycles. The reduction in latent heat compared to pure PEG can be attributed to the constraining effect of the silica matrix, which limits the mobility of PEG molecules within the mesopores. The m-GO-PHSC scaffold maintained substantial thermal energy storage capacity due to the high loading of this PCM. The dramatic photothermal conversion enhancement observed in GO-containing scaffolds is attributed to GO’s high photothermal absorption.^37^ The use of PEG6000 as a control is critical, as its high phase transition temperature (∼66°C) ensures it only functions via sensible heat storage around 41°C, thereby highlighting the unique contribution of latent heat capacity of m-GO-PHSC (PEG1500).^38^ When the temperature approaches its phase transition point (41.2°C), PEG1500 absorbs substantial energy as latent heat, mitigating further heating and providing thermal buffering within a temperature range favorable for osteogenic activity. This thermoregulatory behavior was consistently observed both *in vitro* and *in vivo*, where the m-GO-PHSC (PEG1500) group exhibited slower heating and cooling rates and maintained temperatures within the optimal window, unlike the non-buffering control groups. Together, these findings reinforce the role of the PEG1500-based system as a thermally intelligent scaffold that helps confine temperatures within a range favorable for potential osteogenic activity.

### Biocompatibility, degradation, and osteogenic mechanisms

The m-GO-PHSC scaffold demonstrated excellent biocompatibility and pro-migratory effects *in vitro*, which are critical for facilitating host cell recruitment and tissue integration. The superior cytocompatibility is attributed to the synergistic contributions of the scaffold components: (1) CS, enriched with amino (–NH_2_) and hydroxyl (–OH) groups, promotes cell adhesion and spreading through electrostatic interactions with negatively charged cell membranes,^39^ while also facilitating hydrogen bonding within the network to improve structural cohesion and stability^40^; (2) MSNs provide intrinsically stable and biocompatible interfaces that facilitate cell-material interactions^41^; and (3) PEG microencapsulation within MSNs prevents burst release and minimizes osmotic stress, resulting in a more cell-favorable biochemical environment. The enhanced chemotaxis observed in m-GO-PHSC is likely driven by cooperative interactions between silanol groups on MSNs and cationic CS segments, which collectively increase adsorption of fibronectin and other adhesive proteins, thereby enriching integrin-binding motifs and promoting directed migration.^42^ Although GO-PHSC exhibited comparatively lower activity, it still surpassed the NC group, likely due to its nanoscale topography—which can activate integrin/focal adhesion kinase (FAK)-ERK signaling—and mild electrical conductivity that supports osteogenic cell communication and guidance.^43^ This multifactorial design may offer advantages over simpler systems that often lack such a balanced biochemical and biophysical microenvironment.

The scaffold’s pore architecture is another critical factor influencing its regenerative potential. According to the work by Hulbert et al., smaller pores (10–75 μm) favor soft tissue ingrowth, while pores of 100–200 μm are optimal for bone mineralization, and larger pores promote neovascularization.^44^ The favorable pore size distribution observed in m-GO-PHSC, with 82.9% of pores falling within the ideal 75–200 μm range, can be attributed to the regulatory influence of CS during crosslinking: its functional groups modulate crosslinking behavior and spatial organization,^39^ enabling formation of (1) mid-sized pores for osteogenic differentiation, (2) limited small pores to reduce fibrous encapsulation, and (3) occasional larger pores to facilitate vascular infiltration, while also serving as a structural template that promotes ordered pore architecture and enhances overall scaffold integrity.^45^ The high porosity of 65.4% ± 2.2%, together with the uniform pore architecture, is advantageous for cell infiltration, nutrient transport, and new tissue formation.

The scaffold’s degradation behavior reflects the combined contributions of its components: MSNs gradually hydrolyze into silicic acid through hydroxyl-mediated reactions,^46^ while CS, the major component, undergoes enzymatic and hydrolytic degradation.^47^^,^^48^ Dissolved monosilicic acid can diffuse into surrounding tissues, enter systemic circulation, and be excreted via urine, minimizing long-term accumulation.^49^ These degradation kinetics align well with typical bone healing timelines, suggesting the scaffold’s suitability for *in vivo* regeneration. This was further corroborated by *in vivo* results, which showed controlled degradation without fragmentation and excellent biocompatibility at both local and systemic levels over prolonged implantation.

The *in vitro* and *in vivo* osteogenic results provide preliminary evidence for the scaffold’s bone-regenerative capacity. The scaffold alone (m-GO-PHSC without irradiation) exhibited intrinsic osteoinductive activity. PTT further enhanced bone formation, with more pronounced mineralization in the m-GO-PHSC group. This enhancement may involve two mechanisms. First, the intrinsic activity of the scaffold is likely attributable to sustained Si^4+^ release from degrading MSNs and CS cues^25^; these Si^4+^ ions facilitate HA nucleation/growth,^50^^,^^51^ and may modulate Wnt/β-catenin to upregulate osteogenic markers (ALP, BMP-2, Runx2),^52^^,^^53^^,^^54^ though these signaling events remain speculative and require experimental verification. Second, NIR photothermal stimulation upregulates HSP70, ALP, and BMP-2, while the scaffold’s thermal buffering maintains a favorable temperature. Collectively, the improved bone formation in the PTT group likely results from a combination of the scaffold’s intrinsic properties^25^^,^^55^ and thermal effects, but their relative contributions remain to be determined.

### Conclusion

We developed a CS-modified, form-stable PCM composite photothermal hydrogel (m-GO-PHSC) for bone regeneration. MSNs incorporation stabilized PEG with an encapsulation efficiency of 82.5%, and the resulting PEG@MSNs exhibited a phase transition temperature of 41.2°C and a latent heat of 95.5 J/g. CS optimized scaffold porosity, yielding porosity of 65.4% ± 2.2% with 82.9% of pores in the optimal 75–200 μm range, thereby enhancing both biocompatibility and osteoinductive potential. The hydrogel promoted mBMSC proliferation (cell viability 114.0% ± 7.9% on day 2), chemotaxis (migration rate 1,313.3 ± 40.4 cells at 48 h), and osteogenic differentiation with photothermal treatment showing a trend toward further amplified osteogenic gene expression under the conditions tested. *In vivo*, m-GO-PHSC exhibited favorable degradation without fragmentation and minimal local inflammation, while blood biochemistry and histology confirmed systemic biosafety. Micro-CT and histological analyses demonstrated robust bone formation, with photothermal-treated scaffolds achieving 45.96% ± 2.55% new bone area at 1 month—significantly higher than non-irradiated (15.85% ± 0.93%) and control groups (11.43% ± 0.87%). These results indicate that m-GO-PHSC, combined with localized PTT, may contribute to enhanced bone regeneration and show promise for favorably modulating local tissue responses, highlighting its potential as a multifunctional scaffold for advanced bone tissue engineering.

### Limitations of the study

Several limitations of the present study should be acknowledged. First, the mineralization assay (Alizarin Red S) was performed using scaffold extracts (indirect exposure), whereas the qPCR analysis was performed under direct co-culture on the scaffolds (with or without NIR light). These two exposure models address different biological questions: the former evaluates the intrinsic pro-mineralization capacity of the scaffold material itself, while the latter captures cell–material interfacial interactions and the osteogenic advantage of the phase-change scaffold material during photothermal stimulation. Consequently, the results are complementary rather than directly comparable.

Second, the absence of a composition-matched GO-containing scaffold without PCM precludes the definitive attribution of the enhanced osteogenic effects specifically to PCM-mediated thermoregulation. Although the thermal-buffering capacity of PEG@MSNs was validated by photothermal cycling, physical characterization alone cannot replace biological controls. Whether the observed improvements arise from thermal regulation therefore remains unresolved and warrants further investigation.

Third, this study was conducted in seven-week-old male SD rats, and sex was not analyzed as a biological variable. While the use of a single sex minimizes variability from endogenous sex hormones and is common in initial preclinical evaluations, it limits the generalizability of our findings. Whether the thermoregulatory scaffold exhibits comparable efficacy and safety in female subjects remains to be determined, and future studies with larger cohorts, including both sexes, are warranted to validate the efficacy and safety of this thermoregulatory scaffold.

## Resource availability

### Lead contact

Further information and requests for resources and reagents should be directed to and will be fulfilled by the lead contact, Shuang Pan (panshuang@hrbmu.edu.cn).

### Materials availability

This study did not generate new unique reagents.

### Data and code availability

• All data reported in this paper will be shared by the lead contact upon request.
• This paper does not report original code.
• Any additional information required to reanalyze the data reported will be shared by the lead contact upon request.

## Acknowledgments

This work was supported by the key project of the joint fund of the 10.13039/501100005046Natural Science Foundation of Heilongjiang Province, China (no. ZL2024H004), the Natural Science Foundation of Heilongjiang Province Youth Program (category C, no. QC2026H010), the research funds for Provincial Universities in Heilongjiang Province (no. 2025-KYYWF-ZR0228), the Heilongjiang Provincial Medical and Health Research Project (no. 20250808050519), and open project of the State Key Laboratory of Neurology and Oncology Drug Development (no. SKLSIM-F-2025137).

## Author contributions

Q.Z., conceptualization, investigation, formal analysis, writing – original draft, and funding acquisition; S.Z., investigation and formal analysis; C.S., investigation and data curation; Y.J., investigation and validation; W.Z., data curation, resources, and writing – review and editing; S.P., resources, supervision, funding acquisition, and writing – review and editing.

## Declaration of interests

The authors declare no competing interests.

## STAR★Methods

### Key resources table

**Table:** 

REAGENT or RESOURCE	SOURCE	IDENTIFIER
**Chemicals, peptides, and recombinant proteins**		

Polyethylene glycol (PEG, MW 1500 Da)	Solarbio	Cat#IP9070
Polyethylene glycol (PEG, MW 6000 Da)	Solarbio	Cat#IP9110
Sodium alginate	Aladdin	Cat#S278630
Graphene oxide	XFNANO	Cat#100682
Chitosan	Aladdin	Cat#C105799
DMEM/F12 cell culture medium	Hyclone	Cat#SH30023.01B
Penicillin-Streptomycin	Beyotime	Cat#C0222

**Critical commercial assays**		

CCK-8 Kit	Beyotime	Cat#C0037
2xS6 Universal SYBR qPCR Mix	NovaBio	Cat#Q204-01
Alizarin Red S Staining Kit	Beyotime	Cat#C0148S

**Experimental models: Cell lines**		

Mouse bone marrow mesenchymal stem cells (mBMSCs)	IMMOCELL	N/A

**Experimental models: Organisms/strains**		

SD male rats (7 weeks old)	Beijing Vital River Laboratory Animal Technology Co., Ltd.	RRID: RGD_734476

**Software and algorithms**		

SPSS 26.0	IBM	RRID: SCR_002865
ImageJ software	ImageJ	RRID: SCR_003070

### Experimental model and study participant details

#### Mice

All animal procedures were approved by the Institutional Animal Care and Use Committee (IACUC) of the First Affiliated Hospital of Harbin Medical University (Approval No. IACUC-2024031) and complied with national guidelines and the 3R principles (Replacement, Reduction, and Refinement). Male Sprague-Dawley (SD) rats (7 weeks old; Beijing Vital River Laboratory Animal Technology Co., Ltd.) were housed under controlled conditions (sterile food and water, ∼25 °C). Rats were acclimatized for 7 days prior to experiments. Anesthesia was induced and maintained via isoflurane inhalation.

#### Cell lines

Mouse bone marrow mesenchymal stem cells (mBMSCs) were obtained from IMMOCELL (Xiamen Yimo Biotechnology Co., Ltd., Xiamen, China) and authenticated by flow cytometry. The cells were not tested for mycoplasma contamination in this study. mBMSCs were cultured in DMEM/F-12 complete medium at 37°C with 5% CO_2_. For osteogenic induction, cells under 70-80% confluence were assigned to three models: (1) chemical induction in osteogenic medium, (2) indirect culture with scaffold extracts, or (3) direct co-culture on m-GO-PHSC scaffolds. In the co-culture model, constructs were either subjected to 808 nm NIR irradiation (1.0 W/cm^2^) every 3 days or maintained as non-irradiated controls, with surface temperatures monitored by infrared thermal imaging. Culture medium was refreshed every 72 hours across all groups.

### Method details

#### Raw materials

All reagents were used as received without further purification. Polyethylene glycol (PEG; molecular weights 1500 and 6000 Da, Purity: ≥99%) was obtained from Solarbio (Beijing, China). Sodium alginate (SA, PharmPure™ grade, viscosity 350–550 mPa·s ), chitosan (CS, degree of deacetylation ≥95%, viscosity 100-200 mPa·s), and mesoporous silica nanoparticles (MSNs, ≥99% metals basis) were purchased from Aladdin Reagent (Shanghai, China). Graphene oxide (GO, 2 mg/mL in water, corresponding powder purity 99%) was supplied by XFNANO Materials Technology Co., Ltd. (Jiangsu, China). Mouse bone marrow–derived mesenchymal stem cells (mBMSCs) were obtained from IMMOCELL (Xiamen Immocell Biotechnology Co., Ltd., China). Seven-week-old male Sprague–Dawley (SD) rats were provided by Beijing Vital River Laboratory Animal Technology Co., Ltd. (China) and housed under standard laboratory conditions.

#### Synthesis of mesoporous silica–based PEG form-stable PCMs

A predetermined amount of mesoporous silica nanoparticles (MSNs) was transferred into a conical flask and subjected to vacuum treatment to remove adsorbed gases and surface impurities. Polyethylene glycol was heated in a water bath to its molten state (70 °C) and maintained at this temperature. The molten PEG was then slowly introduced into the pre-vacuumed MSNs system to facilitate uniform infiltration into the mesoporous channels. The mixture was continuously stirred at 70 °C (1000 rpm) for 12 h to ensure homogeneous PEG dispersion within the mesoporous framework. After stirring, the suspension was centrifuged at 10,000 rpm for 10 min to remove excess PEG. The resulting mesoporous silica-based form-stable PCM particles, hereafter referred to as PEG@MSNs, were collected and stored for subsequent use.

#### Preparation of CS-modified form-stable PCM composite photothermal hydrogel

A defined volume of GO solution was transferred into a beaker and diluted to 10 mL with deionized water. The dispersion was ultrasonicated or magnetically stirred at 60 °C (800 rpm) for 1 h to achieve uniform GO distribution. Subsequently, 0.25 g of sodium alginate (SA) and 0.25 g of chitosan (CS) were sequentially added, with each polymer stirred for 1 h to ensure complete dissolution and homogeneous mixing.

Next, 2.0 g of PEG@MSNs particles were introduced, followed by magnetic stirring at 1000 rpm for an additional 2 h to obtain a uniformly dispersed suspension. Gelation was induced by adding 0.1 mol/L CaCl_2_ solution, which effectively immobilized the form-stable PCM particles within the hydrogel network through ionic cross-linking. Finally, the hydrogel was frozen and lyophilized at -80 °C for 36 hours to obtain the CS-modified, form-stable PCM composite photothermal hydrogel. The main sample with PEG1500 is denoted as m-GO-PHSC, while a comparative sample with PEG6000 is labeled m-GO-PHSC(PEG6000). Unless otherwise specified, all subsequent references to m-GO-PHSC in this article pertain to the sample prepared with PEG1500. The control prepared without GO was designated m-PHSC. For comparison, a control hydrogel scaffold was also prepared without CS modification and using free PEG in place of MSNs-encapsulated particles, while keeping the total solution volume and all other preparation steps identical. The resulting phase-change composite hydrogel is designated as GO-PHSC. A schematic illustration of the synthesis procedure and application concept is provided in Figure 2.

#### Thermophysical properties and thermal cycling stability

The phase-transition temperatures and enthalpies of PEG, PEG@MSNs, and m-GO-PHSC were analyzed using differential scanning calorimetry (DSC, NETZSCH, Germany). Measurements were performed under a nitrogen atmosphere at a heating/cooling rate of 5 °C/min within a temperature range of −10 to 60 °C.

Thermal cycling stability was evaluated in a programmable thermal-cycling chamber. Samples underwent repeated heating and cooling between 25 and 70 °C at a rate of 2 °C/min. At both the upper (70 °C) and lower (25 °C) temperature limits, samples were held for 30 min to assess their structural and thermophysical stability over multiple cycles.

#### Photothermal conversion performance

The photothermal performance of the scaffolds, including m-GO-PHSC (with different PEG molecular weights) and the control group m-PHSC, was assessed using the setup illustrated in Figure S1. Specifically, scaffold samples were cut into uniform shapes and placed in a glass container. They were then irradiated with an 808 nm NIR laser at a fixed power density of 1.0 W/cm^2^, with the beam adjusted to fully cover the sample surface. Real-time temperature evolution on the scaffold surface was captured and analyzed using an infrared thermal imaging camera (Testo 865, Germany).

#### Physicochemical and microstructural characterization

Morphological features of the phase-change hydrogel scaffolds were examined by scanning electron microscopy (SEM, SU5000, Hitachi). Freeze-dried samples were sectioned, sputter-coated with gold, and imaged. Pore size distribution was quantified from SEM micrographs using ImageJ. Fourier-transform infrared spectroscopy (FT-IR) was performed using KBr-pellet samples (4000–400 cm^-1^, 0.5 cm^-1^ resolution).

Scaffold porosity was measured via the ethanol-displacement method. A dried sample was placed in anhydrous ethanol with an initial recorded volume (V_1_) and sonicated for 5 min to remove trapped air. The total volume after immersion (V_2_) and the remaining ethanol volume after sample removal (V_3_) were recorded. Porosity was calculated using the formula:

(Equation 1) $$\text{Porosity}\;\left(\%\right)=\left(V_{1}-V_{3}\right)\;/\;\left(V_{2}-V_{3}\right)\times 100\%$$

The *in vitro* degradation behavior of m-GO-PHSC hydrogel scaffolds was evaluated by immersing pre-weighed freeze-dried samples (W_0_, *n* = 3 per time point) in phosphate-buffered saline (PBS, pH 7.4) at 37°C under static conditions. At predetermined time points (0 ∼ 42 days), samples were retrieved, gently rinsed with deionized water, lyophilized, and reweighed (W_t_). The cumulative mass loss was calculated using the formula:

(Equation 2) $$\text{Mass}\;\text{loss}\;\left(\%\right)=\left(W_{0}-W_{t}\right)\;/W_{0}\times 100\%$$

The degradation medium was refreshed every 72 h. All measurements were performed in triplicate, and data were expressed as mean ± SD.

#### CCK-8 cytotoxicity assay

Cytocompatibility of mBMSCs was assessed using a CCK-8 assay. Cells were seeded in 96-well plates (1×10^4^ cells/well) and cultured for 24 h. The medium was replaced with either fresh complete medium (negative control, NC) or scaffold extracts (experimental groups). After 1 and 2 days, 10 μL of CCK-8 solution was added to each well and incubated for 4 h. Absorbance at 450 nm was measured, and cell viability (%) was calculated relative to the NC. Experiments were performed in triplicate and cell viability (%) was calculated as:

(Equation 3) $$\text{Cell}\;\text{viability}\;\left(\%\right)=\left({\text{OD}}_{E}-{\text{OD}}_{B}\right)/\left({\text{OD}}_{\text{NC}}-{\text{OD}}_{B}\right)\times 100\%$$

#### Live/dead cell staining

mBMSCs cultured with scaffold extracts for 48 h were compared with NC cells. Live/dead staining was performed using Calcein-AM (live; green) and propidium iodide (dead; red). Fluorescent images were acquired using a fluorescence microscope (Olympus, Tokyo, Japan).

#### Transwell migration assay

Chemotaxis was evaluated using 24-well Transwell chambers. Lower chambers contained scaffold extracts (experimental) or complete medium (NC). mBMSCs (1×10^5^ cells in 200 μL serum-free medium) were seeded into the upper chambers. After 24 or 48 h at 37 °C, non-migrated cells were removed, and migrated cells on the lower membrane surface were fixed (4% paraformaldehyde), stained with 0.1% crystal violet, and imaged. Migrated cells were quantified from three randomly selected fields per membrane.

#### Alizarin Red S (ARS) staining

P3 mBMSCs were seeded in 12-well plates (2×10^4^ cells/well). At 70–80% confluence, cells were cultured in complete medium (NC), osteogenic medium (OCM), or scaffold extracts in osteogenic medium (experimental). Medium was refreshed every 3 days for 14 days. Cells were fixed with 4% paraformaldehyde, stained with Alizarin Red S, and imaged. Mineralized areas were quantified from three random fields per well.

#### Real-time quantitative PCR (qRT–PCR)

The expression of osteogenesis-related genes in mBMSCs was evaluated during osteogenic induction when co-cultured with m-GO-PHSC scaffolds. Cells cultured in complete medium served as the negative control (NC), while cells in osteogenic differentiation medium served as the positive control (OCM). Experimental groups included mBMSCs seeded on m-GO-PHSC scaffolds with or without NIR irradiation every 72 h. RNA was extracted at days 7 and 14, reverse-transcribed into cDNA, and subjected to quantitative PCR using SYBR Green chemistry. Gene expression was normalized to GAPDH and calculated by the 2^-^ΔΔCT method. Primer sequences are listed in Table 1.

#### *In vivo* degradation and toxicity assessment

Rats were anesthetized and the dorsal surgical site was disinfected with povidone-iodine. An ∼8 mm longitudinal incision was made, and subcutaneous pockets were created for implantation of hydrogel scaffolds, which were sutured in place. After 4 weeks, rats were euthanized and the implants with surrounding tissue were harvested and fixed in 4% paraformaldehyde for analysis.

#### Rat calvarial defect model and NIR irradiation protocol

The rats were randomly divided into three groups using a computer-generated random number table. The investigators performing the surgeries and data analysis were blinded to group allocation. For surgical modeling, the posterior skull region was shaved and disinfected. Anesthesia was induced and maintained with isoflurane. A ∼1 cm coronal incision was made, and the periosteum was gently detached. A 5 mm full-thickness calvarial defect was created using a trephine drill without damaging the dura mater. Cylindrical hydrogel scaffolds (5 mm diameter, 2 mm height) were implanted into the defects, and the periosteum, subcutaneous tissue, and skin were sutured in layers.

For the photothermal groups, NIR irradiation was applied starting on the first day after surgery. An 808 nm NIR laser was used at a power density of 1 W/cm^2^ with a beam diameter of 10 mm, covering the entire defect area. Irradiation was performed once every three days, for 2 minutes per session, under isoflurane anesthesia.

At 4 and 8 weeks post-implantation, rats from each group were euthanized in batches. Calvarial bones were harvested, rinsed with PBS, and fixed in 4% paraformaldehyde for subsequent analysis. Micro-CT and histological evaluations were performed by an operator blinded to the group assignments.

#### Micro-CT scanning and analysis

Rats were euthanized at 1 month or 2 months post-implantation. The harvested samples were fixed, and contrast agents were applied as needed to enhance imaging. Scans were performed using 360° rotational X-ray imaging under optimized scanning parameters to obtain projection data. Reconstruction software was used to generate two-dimensional slices, optimize image quality, and create visualized images. Finally, data were exported for statistical analysis to compare differences among experimental groups.

#### Histological analysis

After fixation in 4% paraformaldehyde, calvarial samples were decalcified in 10–15% EDTA (pH 7.0–7.4) with microwave assistance. The endpoint was confirmed by pinprick test. Following dehydration and paraffin embedding, 4 μm sections were prepared. Sections were stained with hematoxylin and eosin (HE) for general morphology, and Masson’s trichrome for collagen distribution. Images were captured under a light microscope.

### Quantification and statistical analysis

All data were analyzed using SPSS 26.0. Normality of the data was first assessed. For normally distributed data, results are presented as mean ± standard deviation (SD); otherwise, median (interquartile range) was reported. Differences between two groups were assessed using the Student’s t-test, while multiple-group comparisons were performed using one-way ANOVA. A *P*-value < 0.05 was considered statistically significant (∗*P* < 0.05, ∗∗*P* < 0.01, ∗∗∗ *P <* 0.001).
