# Supplementary material for: Thermoregulated photothermal therapy for bone defects using graphene oxide hydrogel with mesoporous silica-derived phase-change nanocapsules
Source: iScience. 2026 Sep 30;29(10):117650. doi: 10.1016/j.isci.2026.117650 (PMC13657222; doi:10.1016/j.isci.2026.117650)
Supplement: Supplementary file 1 [file mmc1.pdf]

iScience, Volume *29*

Supplemental information

Thermoregulated photothermal therapy for bone defects using graphene oxide hydrogel with mesoporous silica-derived phase-change nanocapsules

Qian Zhang, Shuang Zhao, Chenxi Shang, Yanan Jiang, Weiwei Zhang, and Shuang Pan

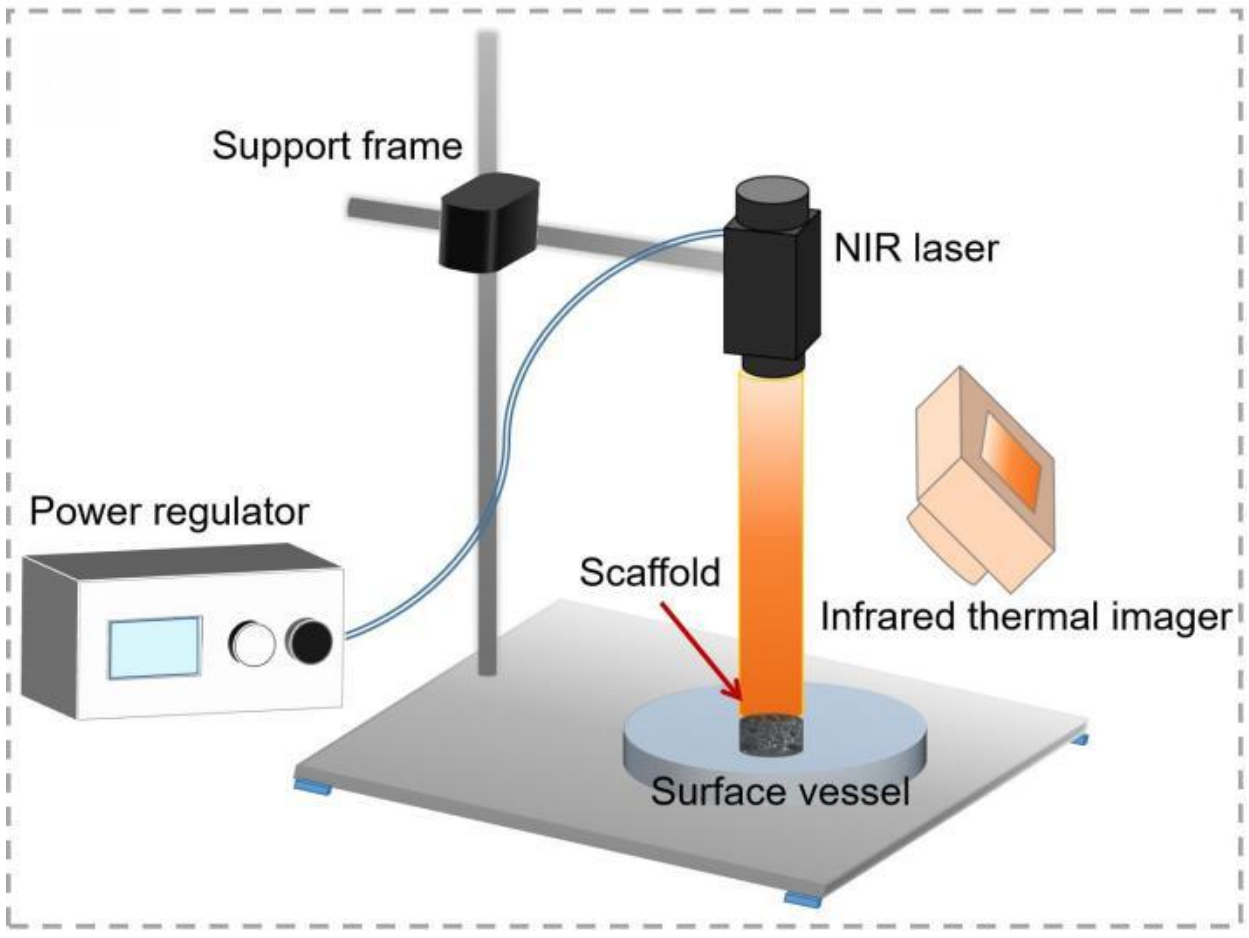

Fig.S1. Schematic of the experimental setup for photothermal conversion measurement. Schematic diagram of the experimental setup for photothermal conversion measurement. Samples were irradiated with an 808 nm NIR laser, and surface temperature was monitored in real-time by an infrared thermal imaging camera

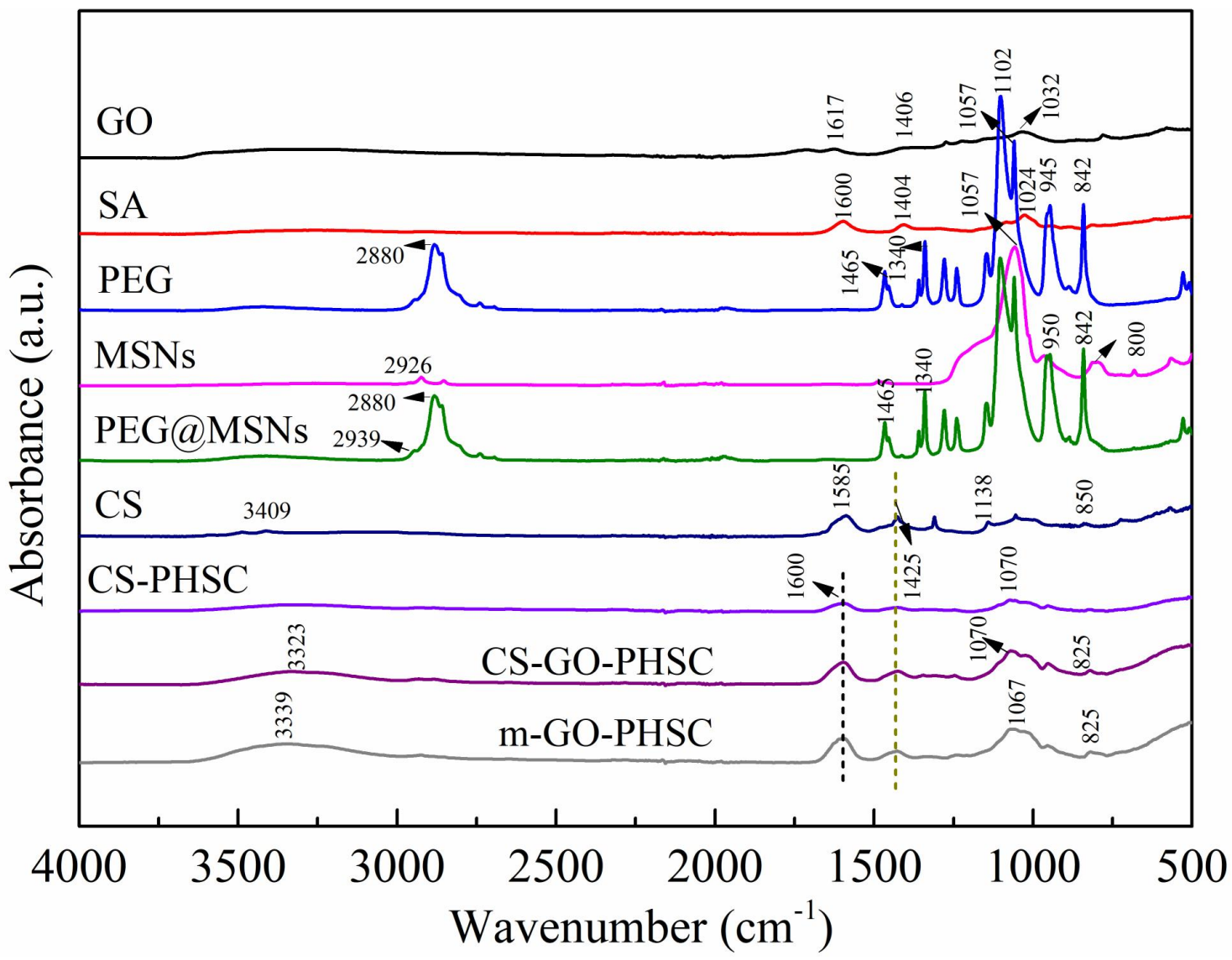

Fig. S2 ATR spectra of m-GO-PHSC hydrogel scaffold and its constituent raw materials. ATR spectra of GO, SA, PEG, MSNs, PEG@MSNs, CS, CS-PHSC, CS-GO-PHSC, and m-GO-PHSC hydrogel scaffolds. Characteristic absorption bands of each component are labeled.

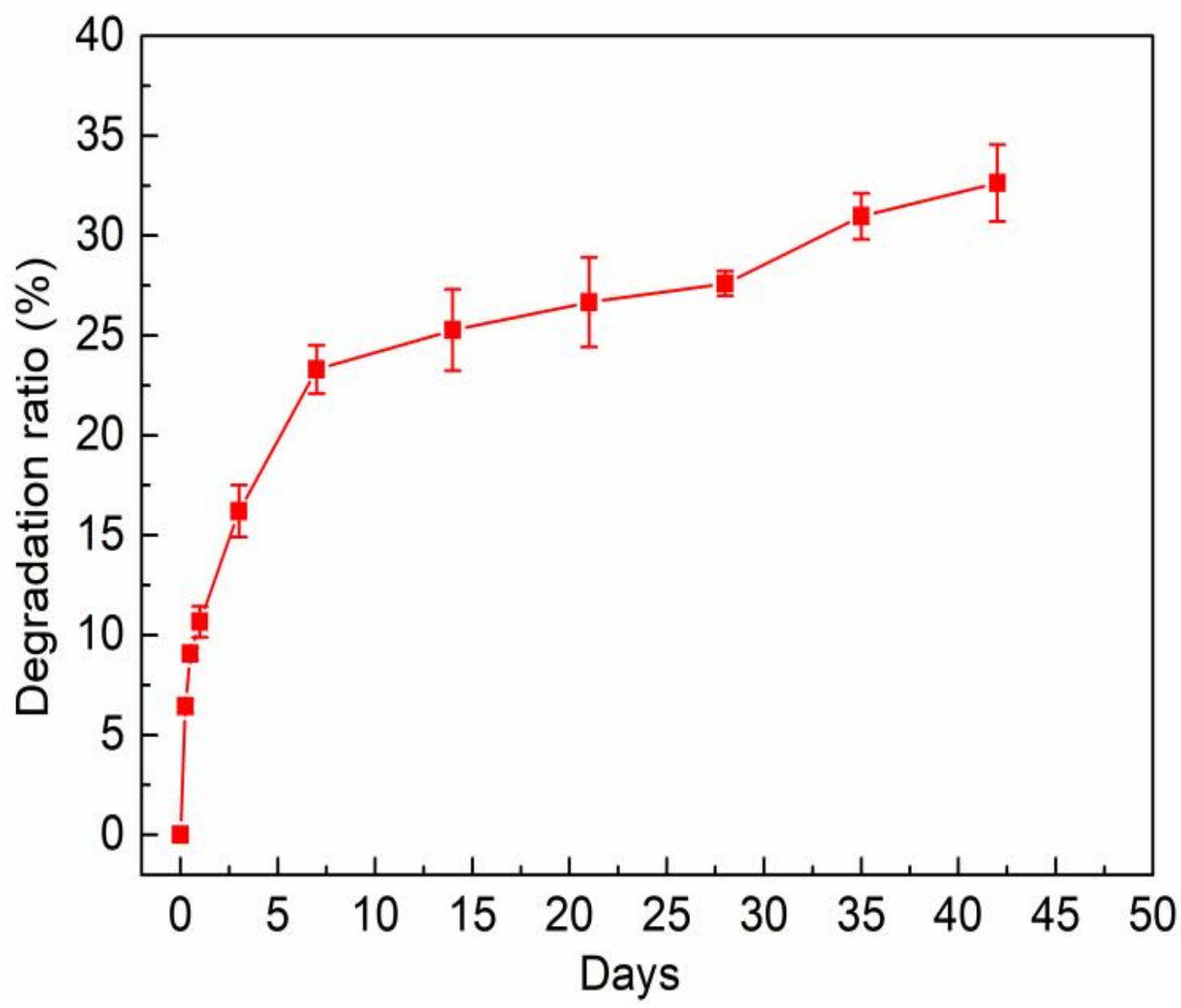

Fig. S3 The in vitro degradation kinetics of m-GO-PHSC hydrogel scaffolds in PBS. In vitro degradation behavior of m-GO-PHSC hydrogel scaffolds. Pre-weighed freeze-dried samples were immersed in PBS (pH 7.4) at 37°C. Cumulative mass loss was measured at the indicated time points over 42 days. Data are presented as mean ± SD (n = 3 per time point).
